# Cyclo[18]carbon Formation from C_18_Br_6_ and C_18_(CO)_6_ Precursors

**DOI:** 10.1021/acs.jpclett.2c02659

**Published:** 2022-10-28

**Authors:** Rahul Suresh, Glib V. Baryshnikov, Artem V. Kuklin, Diana I. Nemkova, Svetlana V. Saikova, Hans Ågren

**Affiliations:** † International Research Center of Spectroscopy and Quantum Chemistry - IRC SQC, Siberian Federal University, 79 Svobodny pr., 660041Krasnoyarsk, Russia; ‡ Laboratory of Organic Electronics, Department of Science and Technology, Linköping University, 60174Norrköping, Sweden; § Division of X-ray Photon Science, Department of Physics and Astronomy, Uppsala University, Box 516, SE-751 20Uppsala, Sweden; ∥ Division of Physical and Inorganic Chemistry, Institute of Non-ferrous Metals, Siberian Federal University, 79 Svobodny pr., 660041Krasnoyarsk, Russia

## Abstract

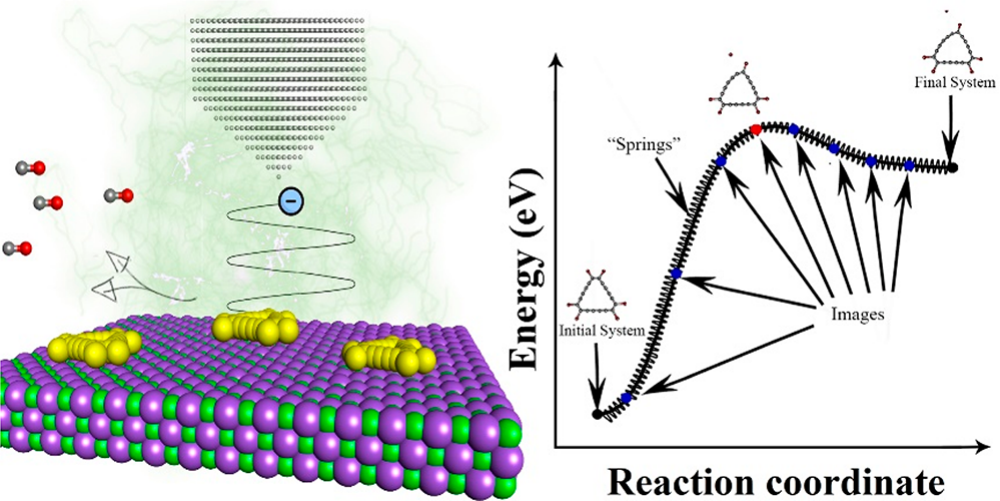

Although cyclo[18]carbon has been isolated experimentally from two precursors, C_18_Br_6_ and C_18_(CO)_6_, no reaction mechanisms have yet been explored. Herein, we provide insight into the mechanism behind debromination and decarbonylation. Both neutral precursors demonstrate high activation barriers of ∼2.3 eV, while the application of an electric field can lower the barriers by 0.1–0.2 eV. The barrier energy of the anion-radicals is found to be significantly lower for C_18_Br_6_ compared to C_18_(CO)_6_, confirming a considerably higher yield of cylco[18]carbon when the C_18_Br_6_ precursor is used. Elongation of the C–Br bond in the anion-radical confirms its predissociation condition. Natural bonding orbital analysis shows that the stability of C–Br and C–CO bonds in the anion-radicals is lower compared to their neutral species, indicating a possible higher yield. The applied analysis provides crucial details regarding the reaction yield of cyclo[18]carbon and can serve as a general scheme for tuning reaction conditions for other organic precursors.

Zaytseva and Neumann^1^ labeled carbon allotropes as “wonder materials” due to their wide potential applications; however, among all the carbon allotropes, the only molecules that have been isolated so far are fullerenes.^2^ Among all the allotropes of carbon, such as carbon nanotubes (CNTs), graphene, graphite, diamonds, and fullerenes, one can assign linear polyynes and carbynes a special significance owing to their fascinating properties.^3,4^

Ring structures of carbon atoms with either alternate single and triple bonds (polyynic) or consecutive double bonds (cumulenic) form a new family of carbon allotropes known as cyclo[*n*]carbons.^5,6^ This allotrope of carbon is now in the limelight among researchers because of its potential applications for various molecular devices.^7−10^ Cyclo[*n*]carbons are observed in the condensed phase, and their properties such as electronic structure, optical spectra, and aromaticity have already been reported using theoretical methods.^11−13^ The cation-radicals of even-number cyclocarbons were recently generated experimentally in the gas phase under special conditions of laser ablation of graphite with subsequent trapping of the target cyclocarbons followed by measurements of their electronic spectra.^14,15^ These data open up possibilities for the astronomical search for cyclocarbons in diffuse clouds by absorption spectroscopy.^15^

Cyclo[*n*]carbons can be synthesized in the gas phase by a process of masking a cyclic precursor and then unmasking it by decomplexation,^16^ retro-Diels–Alder reaction,^6^ cycloreversion,^17^ or decarbonylation.^18^ However, the neutral cyclo[*n*]carbons as individual molecules were not obtained in those works, and only initial precursors and secondary derivatives (like charged ions in mass-spectral experiments) have been possible to detect and characterize.

Recently, another approach was developed in which individual cyclo[*n*]carbons are produced by reactions in an inert atmosphere at low temperatures.^2^ The C_18_(CO)_6_ precursor was deposited on a NaCl bilayer to provide an inert atmosphere at an operating temperature of 5 K, and the experiment was carried out using combined atomic force microscopy (AFM) and scanning tunneling microscopy (STM) systems. The decarbonylate reaction of C_18_(CO)_6_ occurs at an applied bias voltage of +3 V, which leads to the removal of 2, 4, and 6 (−CO) moieties. Later, Scriven et al.^19^ reported the synthesis of cyclo[18]carbon by debromination of C_18_Br_6_ with 5 times more yield than the previous precursor of C_18_(CO)_6_ using the same method and similar conditions but with a low voltage compared to the previous precursor. However, the reaction mechanism and increased yield were not understood properly. This issue can be probed theoretically by employing state-of-the-art quantum chemistry. Understanding the mechanism behind the formation of cyclo[18]carbons from the precursors can be further used to tune the reaction and increase the yield of cyclo[18]carbon.

In this work, we assess the mechanism of cyclo[18]carbon formation in STM experiments by applying an electric field and electron doping to C_18_(CO)_6_ and C_18_Br_6_ molecules which are used as precursors to obtain cyclo[18]carbon. We demonstrate that adding an electron to C_18_Br_6_ results in a substantial lowering of the C–Br dissociation barrier caused by the population of the respective antibonding orbital, while an applied electric field can additionally decrease the energy barrier. This explains the significantly higher yield of C_18_ when C_18_Br_6_ is utilized as a precursor. An analysis of bond length alternation and natural bonding orbitals confirms our results.

We studied the formation of C_18_ from the respective neutral and negatively charged precursors (C_18_Br_6_, C_18_(CO)_6_, and their anion-radicals) by using the nudged elastic band (NEB)^20^ calculation method as implemented in the Vienna Ab initio Simulation Package (VASP).^21−24^ Prior to the NEB calculations, the cells of all initial and final structures were optimized using the PBE functional^25^ and projector-augmented wave (PAW) method.^26^ The weak dispersion interactions were accounted for using Grimme’s D3 correction.^27^ The atomic structures were plotted and visualized using the Vizualisation for Electronic and Structural Analysis (VESTA) software.^28^ The detailed setup can be found in the Supporting Information.

## Structure of C_18_Br_6_ and C_18_(CO)_6_ Precursors on the NaCl Surface

**Figure 1:**
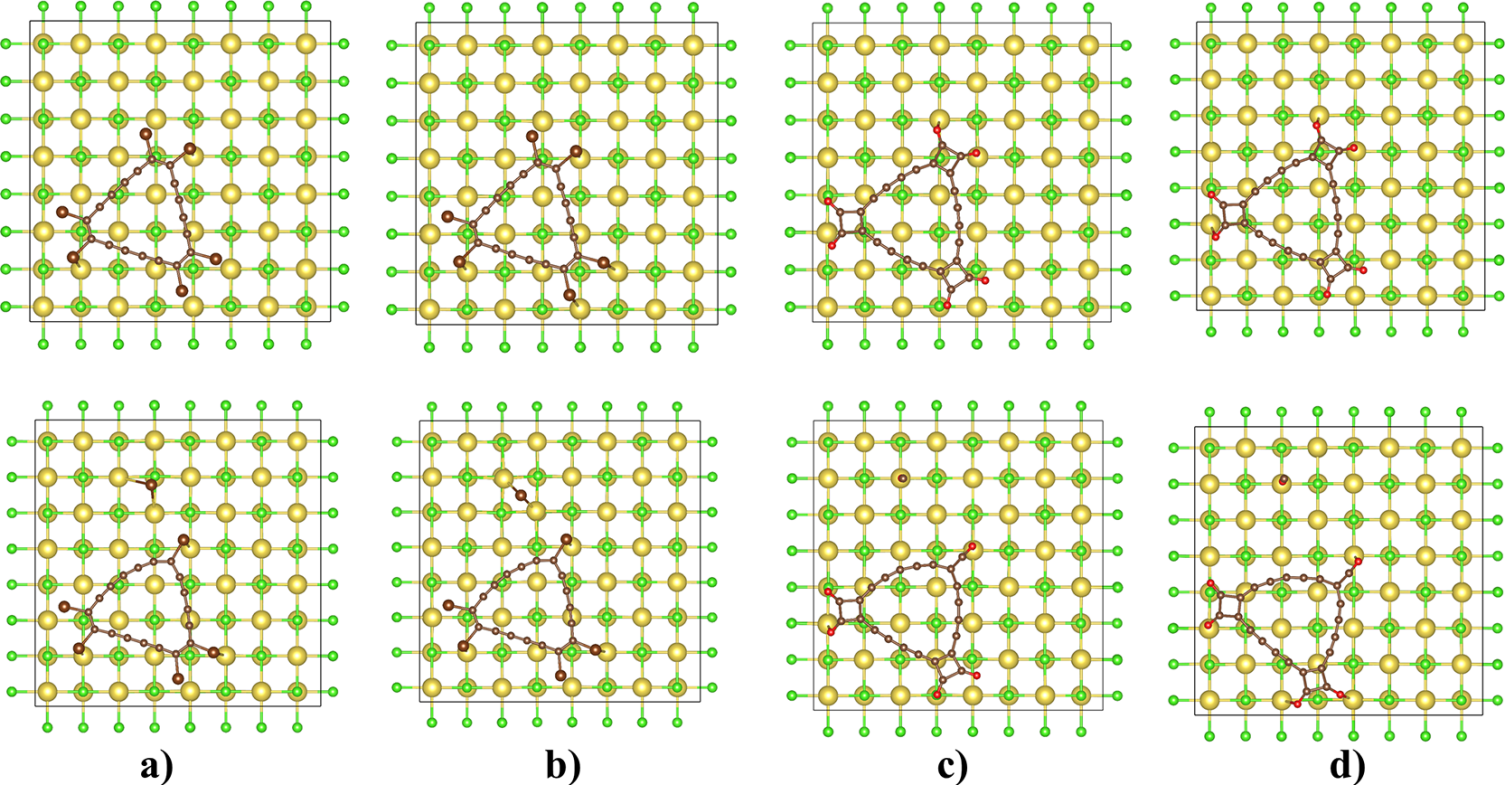
Optimized cells of the initial (top) and final (bottom) structures of the precursors: (a) neutral C_18_Br_6_, (b) anion-radical C_18_Br_6_, (c) neutral C_18_(CO)_6_, and (d) anion-radical C_18_(CO)_6._.

The C_18_Br_6_ and C_18_(CO)_6_ precursor molecules are initially optimized assuming planar structures (Figure S1) before placing them on the NaCl bilayer which is used in the experimental synthesis as a substrate. The precursor molecules have a similar structure where the C_18_ component is aligned in a triangular shape with alternative triple and single bonds and with the carbon atoms in the corners being bound with two −Br and −CO moieties (Figure 1). The C_18_Br_6_ and C_18_(CO)_6_ molecules are initially placed in different positions on the NaCl surface at the distance of ∼3.30 Å between the surface and the precursor molecule and then optimized. The most energetically favorable position is adopted for further calculations. The C_18_Br_6_ molecule does not undergo any significant structural changes during optimization, whereas the C_18_(CO)_6_ molecule loses the planarity of the precursor molecule and is distorted due to the interaction with the surface. This finding is in fair agreement with the results of atomic force microscopy.^2^ The structure becomes nonplanar, and the distance to the NaCl surface varies from 3.1–3.5 Å.

In order to simulate the dissociated state, the −Br and −CO species were placed at a sufficient distance from the precursor molecule to avoid any spurious interactions, and then an energy minimum was found. From the optimization results of the final structure of the precursors, one can see that the C_18_ core loses its linear alignment on dissociation of the −Br atom and the −CO molecule while the bond order of the core structure remains the same. The dissociated Br atom settles above the Cl atom of the surface in the case of the neutral system, and for the anion-radical system, the Br atom settles above the interstitial space between the Na and Cl atoms (Figure 1). The average distance between the surface and the Br atom is larger than 2.70 Å. In the case of C_18_(CO)_6_, for both neutral and anion-radical systems, the −CO molecule settles above the Na atom with a distance larger than 2.60 Å. Bader charge analysis^29,30^ was carried out to understand the charge transfer between the surface and the dissociating molecule. The dissociated Br atom gains a charge of −0.34 and −0.48 *e* for the neutral and anion-radical systems, but the charge transfer from the surface to the CO molecule is negligible because the oxygen atom is bound with the NaCl surface and stabilized by the carbon atom.

## Debromination and Decarbonylation of C_18_Br_6_ and C_18_(CO)_6_

**Figure 2:**
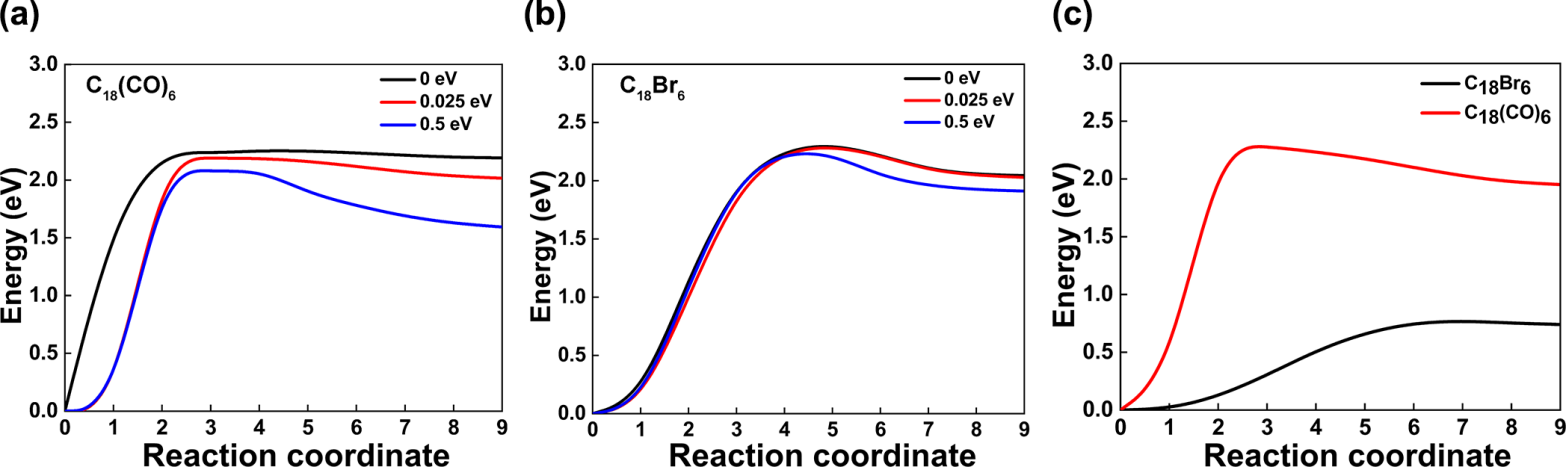
Reaction path from NEB calculation for C_18_Br_6_ and C_18_(CO)_6_. (a) NEB plot for C_18_(CO)_6_, (b) NEB plot for C_18_Br_6_, and (c) NEB plot for negatively charged (anion-radical) systems. The reaction coordinate refers to the images interpolated between the initial (ground) state “0” and the final state “9”. Each intermediate image is minimized in energy along the reaction path while keeping equal spacing with neighboring images.

The mechanism behind the experimental yield of cyclo[18]carbon from the different precursors is determined by the NEB calculations. The adsorption site of the precursor molecules on the NaCl surface is defined by performing structural relaxation for several possible orientations, and the minimum energy structure is so used for the NEB simualtions. The distance between the dissociated atom/molecule from the initial structure of C_18_Br_6_ and C_18_(CO)_6_ is 5.55 and 6.01 Å, respectively. The NEB setup was constructed by interpolating 8 images from the final state, which is the dissociated configuration of the precursor molecule. The first step was additionally interpolated by 8 images to increase the accuracy of the dissociation. The relation between the reaction coordinate and the energy is plotted as graphs shown in Figure 2.

## Debromination and Decarbonylation of Neutral C_18_Br_6_ and C_18_(CO)_6_

The elimination of a single Br atom and CO molecule at a time from their respective precursors is demonstrated here. For both the precursor molecules, a saddle point is observed at an energy barrier of ∼2.3 eV for the systems without an input voltage. However, with an input voltage of 0.5 eV, the energy barrier is further reduced. It is evident that the reaction at 0.5 eV input voltage has less activation energy compared to the system with an input voltage of 0 and 0.025 eV, which confirms the necessity of an external electric field for the reaction to take place effectively. We found that the Br atom and CO molecule have to overcome an energy barrier of ∼2.3 eV to be dissociated from their precursor molecules in the absence of an external field. Upon the applied electric field of 0.025 eV, there is a very slight variation in the energy barrier for both the precursor molecules, but with a 0.5 eV applied electric field, the energy barrier decreases to ∼2.20 eV for C_18_Br_6_ and ∼2.1 eV for C_18_(CO)_6_. Thus, the voltage applied to the STM tip itself can reduce the reaction barrier by inducing an electric field.

## Debromination and Decarbonylation of Negatively Charged [C_18_Br_6_]^−^ and [C_18_(CO)_6_]^−^

For further understanding, we have calculated the energy barrier for the anion-radical systems using NEB (Figure 2c). The saddle point for [C_18_Br_6_]^−^ debromination is observed at an energy barrier of 0.75 eV and at 2.25 eV for C_18_(CO)_6_, which indicates that the Br atom and CO molecule have to overcome an energy barrier of 0.75 and 2.25 eV, respectively, for the bond dissociation to occur. Overall, there is no significant difference in the energy barrier for the bond dissociation to occur between the neutral and anion-radical systems of C_18_(CO)_6_, whereas the negatively charged C_18_Br_6_ precursor molecule is highly affected toward C–Br bond dissociation. This explains the five times higher reaction yield for debromination of C_18_Br_6_ (64% in ref (19)) compared to C_18_(CO)_6_ (13% in ref (2)) at similar conditions. It is also in agreement with the energetics of a synergistic mechanism or CO elimination from C_18_(CO)_6_ demonstrated in the previous theoretical research,^31^ showing that the reaction cannot happen in ambient conditions because of the high energy barrier of around 2.1 eV which thus is comparable to our NEB result (∼2.25 eV). The drastic decrease in energy for debromination of [C_18_Br_6_]^−^ can be associated with the population of the LUMO orbital of C_18_Br_6_ by one electron. This orbital demonstrates an antibonding character toward the C–Br orbital (an additional discussion of this issue can be found in the next section). The saddle point for [C_18_Br_6_]^−^ debromination shifts to reaction coordinate 7 (Figure 2c, black line), reflecting an easy C–Br bond dissociation (reaction coordinates 0–5). The new barrier is mostly associated not with the bond dissociation, but with the diffusion of the species on the NaCl surface.

Therefore, we demonstrated that the C_18_(CO)_6_ precursor could be mostly affected by the electric field generated by STM, while C_18_Br_6_ could have high sensitivity to the electron tunneling effect induced by the STM tip. Our theoretical predictions confirm the experimental statement that the cyclo[18]carbon yield from the C_18_Br_6_ precursor is much higher than from C_18_(CO)_6_^19^ and that this difference might be because the precursor molecule exists in a charged state and hence less energy is required for the bond dissociation to occur. One additional conclusion can be obtained from the analysis of the [C_18_Br_6_]^−^ debromination reaction profile: the reverse bromination reaction is almost barrierless and thermodynamically favorable leading to the fact that releasing Br atoms by forming Br_2_ molecules is important for the efficient generation of cyclo[18]carbon.

## Electron Localization Function Analysis

**Figure 3:**
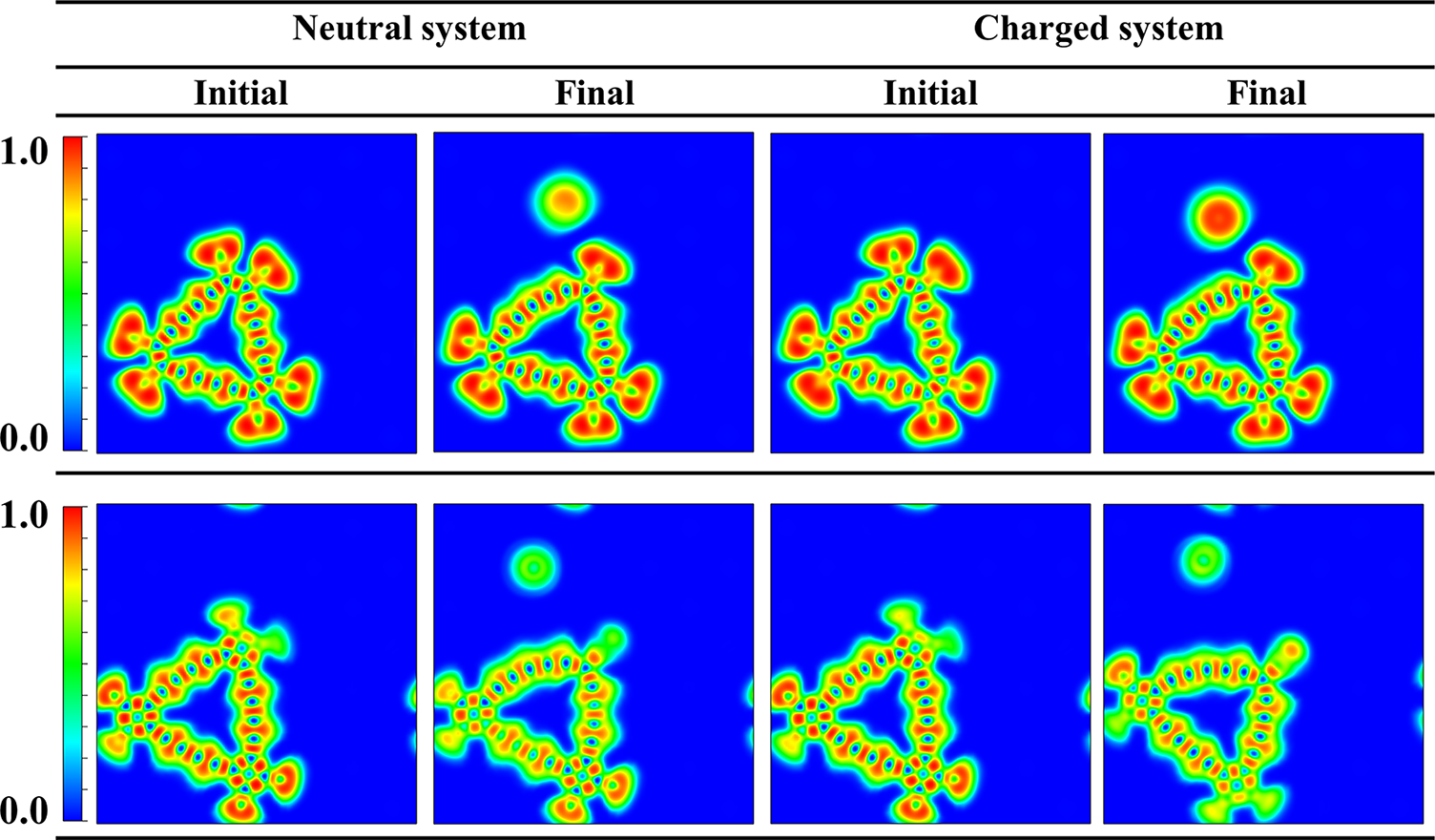
Electron localization function (ELF) of the initial and final states for both neutral and charged (anion-radical) C_18_Br_6_ (top) and C_18_(CO)_6_ (bottom) renormalized between 0.0 and 1.0. Red corresponds to fully localized electrons, and green corresponds to fully delocalized electrons.

For the topological understanding of the system, we have calculated the electron localization function (ELF) for the initial and final states of the precursor molecule^32−34^ (Figure 3). The ELF gives the necessary information about the atomic interactions, electron pairs, location of bonds, and the strength of the bonds which can be displayed as an electron density map.^35^ The ELF value is here represented as a contour map in which green refers to a delocalized state and red corresponds to a fully localized state. To investigate the contour pattern to distinguish the chemical bonding from physical bonding, the ELF is plotted with the charge integration over the basin for the different systems. The ELF distribution is different for different bond types where both precursor molecules exhibit highly localized electron density between the C–C bonds. The 2D shape of the ELF between the C–C bond resembles a torus shape and a spherical shape, which is the characteristic distribution of a triple bond and single σ bond.

For the final state of the precursor molecules, there is a high localization of the electron density around the anion which is spherical in shape, but the electron density for the same atoms is also distorted in the initial structure. However, the ELF distribution of the dissociated Br demonstrates more pronounced electron localization features, revealing that the extra electron added to the system could reside on the dissociated species. The ELF distribution for C_18_(CO)_6_ is not uniform considering the nonplanarity of the structure, but it can be observed that the electron density distribution is the same for both precursor molecules. The red region around Br/CO corresponds to the electron pairs that are fully localized, whereas the green region is distributed among the C_18_ framework which is responsible for the aromaticity of the molecule. The 2D ELF plots directly demonstrate that the precursor molecules are not in a perfectly flat configuration on the NaCl surface, which is in fair agreement with experimental AFM observations. In order to visualize the excessive charge effect in the anion state, we calculated the charge density difference plot between the anionic and neutral states of the precursor molecules (Figure S2). Comparing the charge redistribution between the final state of C_18_Br_6_ and C_18_(CO)_6_, the plot shows significantly more charge distribution on the isolated Br than on the isolated CO.

## Aromaticity of C_18_Br_6_ and C_18_(CO)_6_ Molecules and Their Charged States

**Figure 4:**
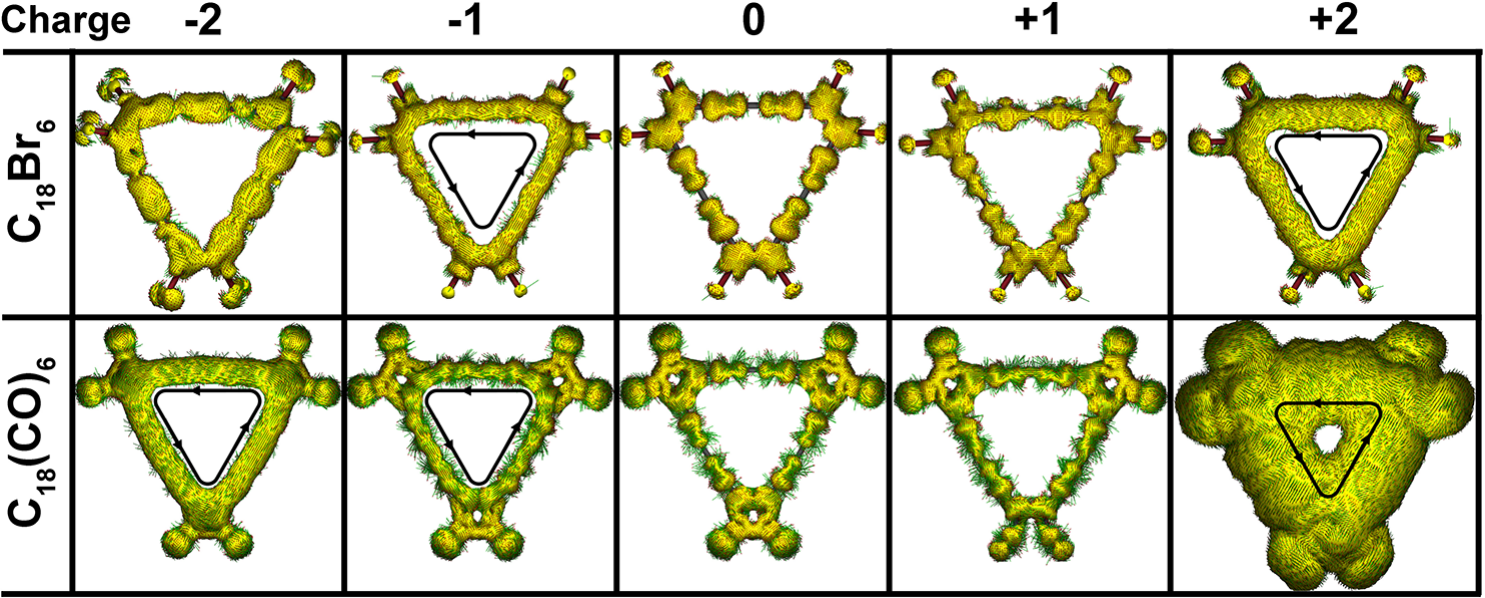
AICD plots for the C_18_Br_6_ and C_18_(CO)_6_ molecules and their charged states. The black arrows represent the direction of the induced current. The direction of the magnetic field is perpendicular to the plane of the molecules. The isosurface value is set to 0.05 au.

In order to understand the aromaticity of the precursors in the neutral and charged states, we have optimized C_18_Br_6_ and C_18_(CO)_6_ using density functional theory (DFT)^36^ at the wB97XD/6-311g(d,p)^37,38^ level of theory for different charges (−2, −1, 0, +1, +2) using Gaussian 16,^39^ and the optimized structures are reported in the Supporting Information. The wB97XD range separated hybrid functional reproduces well the experimental structures of the neutral C_18_Br_6_ and C_18_(CO)_6_ species^2,19^ (Figure S1), which also are in agreement with previous theoretical results.^31,40^ Some deviation in angles of C_18_Br_6_ can be explained by the fact that the experimentally detected precursor is not flat, something that could be the result of surface effects. From Figure S3 it can be seen that the structures remain planar in all cases except for −2 charged C_18_Br_6_ and +2 charged C_18_(CO)_6_ which are deformed from their planar nature. Here we would like to focus mainly on the structural deviations that occur by adding an electron to the C_18_Br_6_ and C_18_(CO)_6_ molecules. The single–triple bond length alternation (BLA) for C_18_Br_6_ and C_18_(CO)_6_ species versus their anion-radicals is very similar by magnitude but smaller by 0.01 Å in average for charged systems; the C–C bonds adjacent between the four-membered rings and the C_18_ frame also changed; two of them are much longer in the anion-radical state in comparison to the neutral one (1.416 vs 1.370 Å). The BLA parameter also tends to decrease slightly for the anion-radical of C_18_Br_6_ compared to the neutral system and similarly to C_18_(CO)_6_. The C–Br bonds elongate by 0.015–0.025 Å following the antibonding character of the LUMO orbital of C_18_Br_6_ toward the C–Br bond (it becomes a single-occupied orbital in the anion-radical state). The attachment of one extra electron to the C_18_Br_6_ and C_18_(CO)_6_ molecules makes them clearly antiaromatic as follows from the plots of anisotropy of the induced current density (AICD); both [C_18_Br_6_]^−^ and [C_18_(CO)_6_]^−^ anion-radicals sustain the anticlockwise magnetically induced currents within the C_18_ frame, while neutral C_18_Br_6_ and C_18_(CO)_6_ compounds are nonaromatic species (Figure 4). Thus, single-electron attachment to C_18_Br_6_ and C_18_(CO)_6_ molecules makes the resulting anion-radicals antiaromatic, which transform into the doubly aromatic C_18_ state by six-time decarbonylation/debromination. Elongation of the C–Br bonds in the [C_18_Br_6_]^−^ anion-radical is an additional precondition for loosing Br atoms. Attachment of the second electron to the [C_18_Br_6_]^−^ and [C_18_(CO)_6_]^−^ anion-radicals induces a structural out-of-plane distortion and loss of antiaromatic behavior for the resulting [C_18_Br_6_]^2–^ (Figure 4), while [C_18_(CO)_6_]^2–^ remains planar and sustains even stronger paratropic currents within the C_18_ frame (i.e., more antiaromatic) than the [C_18_(CO)_6_]^−^ anion-radical.

One-electron oxidation of C_18_Br_6_ and C_18_(CO)_6_ until the cation-radical state does not significantly change their nonaromatic character: one can observe some small diatropic current for [C_18_(CO)_6_]^+^ and even smaller paratropic current for the [C_18_Br_6_]^+^ species. Further oxidation of C_18_Br_6_ and C_18_(CO)_6_ molecules until a dicationic state induces the appearance of strong paratropic currents within the C_18_ perimeter similar to the C_18_(CO)_6_ dianion. This is in agreement with the [4*n*] Hückel’s rule for antiaromaticity (*n* = 4 for dications and *n* = 5 for dianions, while the C_18_Br_6_ dianion is nonplanar due to the twisted structure; Figure 4).

## Electronic Properties of the C_18_Br_6_ and C_18_(CO)_6_ Molecules and Their Anion-Radical States

**Figure 5:**
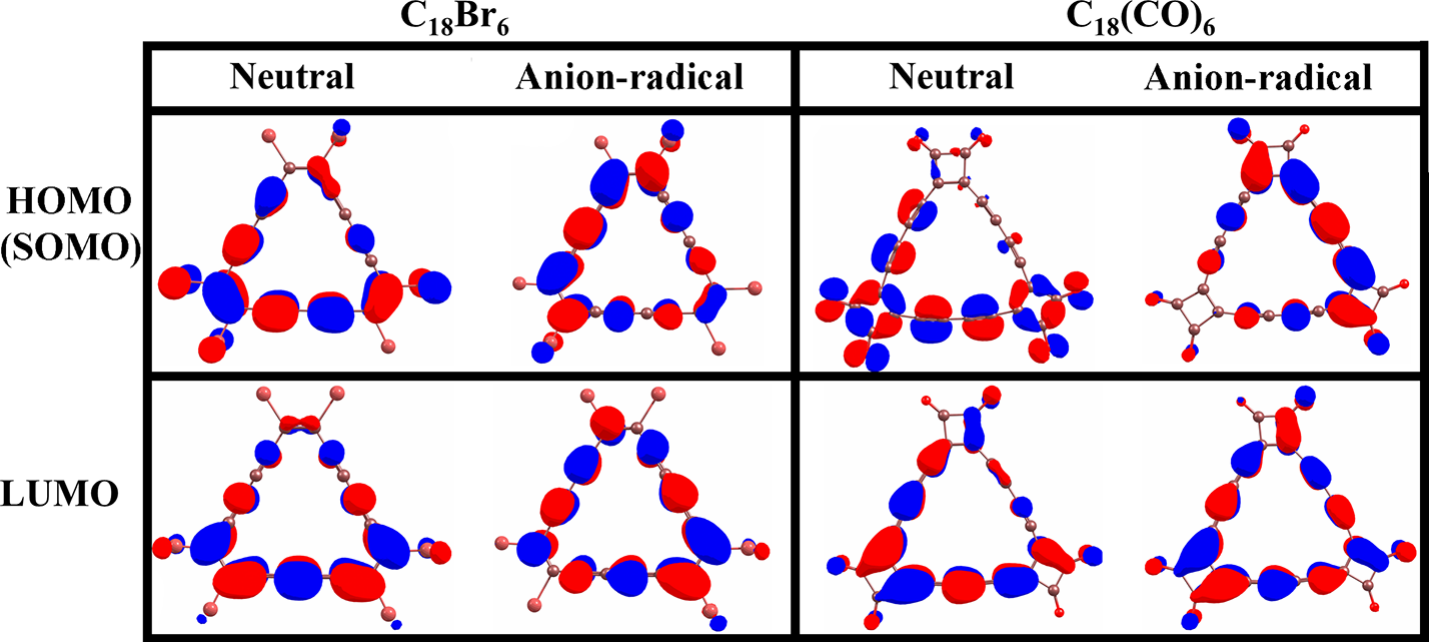
Localization of HOMO/SOMO and LUMO states in neutral and charged C_18_Br_6_ and C_18_(CO)_6_. The α spin state is considered for both HOMO/SOMO and LUMO levels.

To understand the chemical reactivity of the molecules, we have performed a frontier molecular orbital analysis. The localizations of the highest (or singly) occupied molecular orbitals (HOMO/SOMO) and lowest unoccupied molecular orbitals (LUMO) in spin-alpha states are shown in Figure 5. The orbitals are localized primarily within the C_18_ frame for both neutral and anion-radical systems. However, the HOMO and LUMO levels show a significant amount of energy change between the neutral and anion-radical systems (Table 1). The HOMO and LUMO levels of C_18_Br_6_ are found to be −8.31 and −1.81 eV, respectively, and for C_18_(CO)_6_, the HOMO and LUMO levels are −9.32 and −3.1 eV, respectively. The SOMO and LUMO levels of the anion-radical precursors are shifted to higher energies, −2.87 and 1.2 eV and −4.06 and −0.2 eV, respectively. The energy gap in the anion-radical C_18_Br_6_ system is found to be −4.07, which is 2.43 eV smaller than in the neutral system. Likewise, in the case of the anion-radical C_18_(CO)_6_ system, the energy gap is found to be 3.86 eV, which is 2.36 eV smaller than in the neutral system.

**Table 1:** Computed HOMO/SOMO, LUMO, Energy Gap (*E*_g_), and Global Indices (in eV) for Neutral and Anion-Radical C_18_Br_6_ and C_18_(CO)_6_

system	HOMO (SOMO)	LUMO	energy gap	χ	η	ω
C_18_Br_6_ - neutral	–8.31	–1.81	6.50	5.06	3.25	3.93
C_18_Br_6_ - anion-radical	–2.87	1.20	4.07	0.84	2.04	0.17
C_18_(CO)_6_ - neutral	–9.32	–3.10	6.22	6.21	3.11	6.20
C_18_(CO)_6_ - anion-radical	–4.06	–0.20	3.86	2.13	1.93	1.17

We have employed Koopmans’ theorem^41^ to realize the electrophilic and nucleophilic behavior of the precursor molecules which will further indicate the reactivity of the molecule with respect to charge. The global indices of electronegativity (χ), hardness (η), chemical potential (μ), and the electrophilicity index (ω) are calculated using the following formula:^42^

Electronegativity is a measure of efficiency of a molecule to attract electrons toward itself
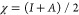
Chemical hardness is the measure of resistance of a molecule to charge transfer
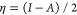
Chemical potential is the tendency of an electron to escape from equilibrium
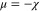
Electrophilicity index is the measure determining the nature of a molecule to accept electrons
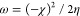
where *I* and *A* are the ionization potential and electron affinity, respectively. From the values of the global indices listed in Table 1, it is very evident that the neutral molecules possess more affinity to accept electrons compared to the anion-radical system with a maximum electrophilicity index of 6.20 eV for neutral C_18_(CO)_6_. The electrophilicity index also corresponds to the stability of the system^43,44^ by the relation “the larger the value, the lower the reactivity”, making the system more stable. The larger value for C_18_(CO)_6_ indicates the increased stability of the molecule and hence a requirement of higher energy for a reaction to occur. As the experimental evidence states a higher yield for the C_18_Br_6_ debromination reaction, there is a high probability that the electrophilicity index as well as vertical electron affinity directly correlate with the higher reactivity of C_18_Br_6_ toward C–Br dissociation rather than C–CO dissociation by C_18_(CO)_6_.

It is important to consider the bond stability and intermolecular interactions in the precursor molecule to understand the possibility of bond dissociation. Here, we have applied a second-order perturbation method incorporated in the natural bonding orbital analysis (NBO) of Gaussian 16 to calculate the bond type and the stabilization energy *E*^(2)^. This stabilization energy is expressed as a relation of electron delocalization between donor and acceptor atoms and is calculated using the formula
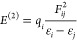
where *q*_i_ is the donor orbital occupancy, ε_i_ and εj are diagonal elements of orbital energies, and *F*_*ij*_ is the Fock matrix element from the NBO.^45^

The prominent bonding (BD) to antibonding orbital (BD*) transitions are tabulated in Table 2. It can be observed that C–C bonds in the C_18_ ring show higher stability than the C–Br bonds, while for the C_18_(CO)_6_ system, the C–CO bonds are highly stabilized making it more difficult to be dissociated compared to C_18_Br_6_. Likewise, for anion-radical systems, the stability of C–Br and C–CO bonds is comparatively smaller than their respective neutral systems, indicating the possibility of a higher yield from the anion-radical systems.

**Table 2:** Second-Order Perturbation Analysis Showing the Prominent Charge Transfer Interactions in C_18_Br_6_, Anion-Radical C_18_Br_6_, C_18_(CO)_6_, and Anion-Radical C_18_(CO)_6_^a^

donor NBO	acceptor NBO	interaction type	*E*^(2)^(kcal/mol)
Neutral C_18_Br_6_			
C1–C2	C3–C4	BD → BD*	22.51
C1–C2	C17–C18	BD → BD*	20.75
C5–C6	C7–C8	BD → BD*	21.13
C1–C18	C17–Br22	BD → BD*	6.27
C4–C5	C6–Br20	BD → BD*	6.25
C15–Br19	C3–C4	BD → BD*	2.15
Anion-Radical C_18_Br_6_			
C1–C2	C17–C18	BD → BD*	12.57
C3–C4	C1–C2	BD → BD*	18.24
C6–C8	C7–C9	BD → BD*	18.17
C7–C9	C10–C11	BD → BD*	19.09
C6–Br20	C7–C8	BD → BD*	2.95
Neutral C_18_(CO)_6_			
C1–C2	C3–C4	BD → BD*	21.40
C1–C2	C17–C18	BD → BD*	23.78
C5–C6	C23–O29	BD → BD*	25.32
C 5-C6	C24–O30	BD → BD*	25.30
Anion-Radical C_18_(CO)_6_			
C1–C2	C3–C4	BD → BD*	10.92
C3–C4	C5–C6	BD → BD*	12.38
C5–C6	C23–O29	BD → BD*	14.32
C7–C8	C9–C10	BD → BD*	11.17
C18–C22	C21–O27	BD → BD*	2.73

a The atom numbers correspond to those represented in Figure S1.

In summary, using first-principles calculations, we explored the mechanism of formation of a new carbon allotrope known as cyclo[18]carbon from the C_18_Br_6_ and C_18_(CO)_6_ precursor molecules. We revealed the dissociation barrier energy for C–Br and C–CO bonds with and in the absence of an electric field, showing the role of the field in lowering the barriers. It was found that there is no significant change in the barrier energy of an anion-radical C_18_(CO)_6_, whereas for C_18_Br_6_, the barrier energy drastically falls (to 0.75 eV), which indicates a higher yield of cyclo[18]carbon as predicted by a previous experimental study. The bond length alternation parameter shows elongation of the C–Br bond length that turns out to be a precondition for the bond dissociation to occur, which is confirmed further by a natural bonding orbital analysis. Global indices are analyzed and calculated for the precursor molecules and their anionic states, showing that the anionic state of C_18_Br_6_ is highly reactive compared to the other systems. While the neutral C_18_Br_6_ and C_18_(CO)_6_ species are nonaromatic, both C_18_Br_6_ and the C_18_(CO)_6_ anion-radicals demonstrate antiaromaticity sustaining anticlockwise magnetically induced currents within the C_18_ frame. This work demonstrates that computational chemistry can provide great detail and explanation of the efficiency and selectivity of reactions creating cyclo[18]carbon. The scheme of analysis put forward in this work can probably be applied also to other carbon allotropes formed from precursor molecules.

## Supporting Information Available

Computational details, structural parameters of neutral and charged C_18_Br_6_ and C_18_(CO)_6_ precursor molecules, and charge density difference map of both initial and final state of precursor molecules reflecting the difference between neutral and anion-radical states (PDF)
Transparent Peer Review report available (PDF)
