# Supplementary material for: Cyclo[18]carbon Formation from C18Br6 and C18(CO)6 Precursors
Source: J Phys Chem Lett. 2022 Oct 28;13(44):10318–25. doi: 10.1021/acs.jpclett.2c02659 (PMC9661529; doi:10.1021/acs.jpclett.2c02659)
Supplement: Supplementary file 1 — jz2c02659_si_001.pdf [file jz2c02659_si_001.pdf]

Supporting information for

Cyclo[18]carbon Formation from $C_{18}Br_6$ and $C_{18}(CO)_6$ Precursors

Rahul Suresh[1], Gleb V. Baryshnikov[2,*], Artem V. Kuklin[3,*], Diana I. Nemkova[1,4], Svetlana V. Saikova[4] and Hans Ågren[3]

[1]*International Research Center of Spectroscopy and Quantum Chemistry - IRC SQC, Siberian Federal University, 79 Svobodny pr., 660041 Krasnoyarsk, Russia*
[2]*Laboratory of Organic Electronics, Department of Science and Technology, Linköping University, 60174, Norrköping, Sweden*
[3]*Department of Physics and Astronomy, Uppsala University, Box 516, SE-751 20 Uppsala, Sweden*
[4]*Division of Physical and Inorganic Chemistry, Institute of Non-ferrous Metals, Siberian Federal University, 79 Svobodny pr., 660041 Krasnoyarsk, Russia*

Computational details

We studied the formation of C_{18} from the respective neutral and negatively charged precursors ($C_{18}Br_6$, $C_{18}(CO)_6$ and their anion-radicals) by using the Nudged Elastic Band (NEB)[1] calculational method as implemented in the Vienna Ab initio Simulation Package (VASP)[2–5] to identify the activation energy barrier, where 8 images were constructed between the initial and final configurations. The whole calculation is set up on a bilayer NaCl surface with vacuum space of 14 Å along the non-periodical direction to avoid interactions between the adjacent unit cells due to the periodic boundary conditions. The NEB calculations are carried out with a spring constant of -5 eV/Å^{-2} and a scaling constant of 0.02 with fixed volume and only the atomic positions are allowed to relax during the calculations using the conjugate gradient algorithm. Prior to the NEB calculations, the cells of all initial and final structures were optimized using the PBE functional[6] proposed by Perdew, Burke, Ernzerhof (PBE) to avoid spurious relaxations during the determination of the saddle point. The calculations for initial and final configurations of both $C_{18}Br_6$ and $C_{18}(CO)_6$ were carried out at different values of an applied electric field to complement the findings from the STM results. The ion-electron interactions were calculated using projector-augmented wave (PAW) potentials and a plane wave energy cutoff of 400 eV was employed.[7] All structures were optimized until the force of each atom converged to 0.01 eV/Å. The convergence criterion for the energy was set to 10^{-4} eV. The weak dispersion interactions between the precursor molecules and the NaCl surface were accounted by using Grimme's D3 correction inorder to understand if the NaCl surface includes any considerebale effect on the adsorption of the precursor molecules.[8] The atomic structures were plotted and visualized using the Electronic and Structural Analysis software (VESTA).[9]

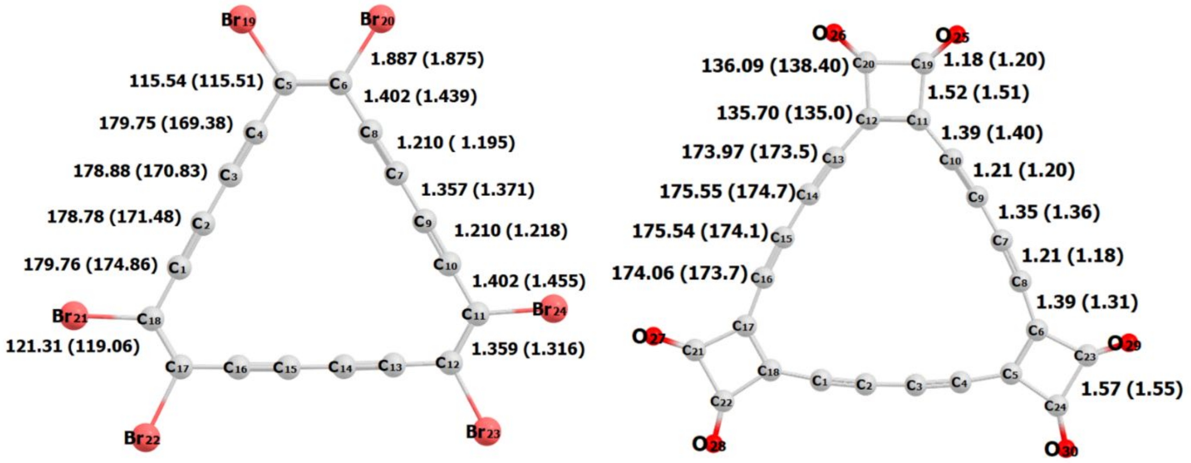

Figure S1: Calculated and experimental (in parenthesis) structural parameters of neutral $C_{18}Br_6$ (left panel) and $C_{18}(CO)_6$ (right panel). The labels on the left indicate the bond angles and labels on the right indicate the bond lengths.

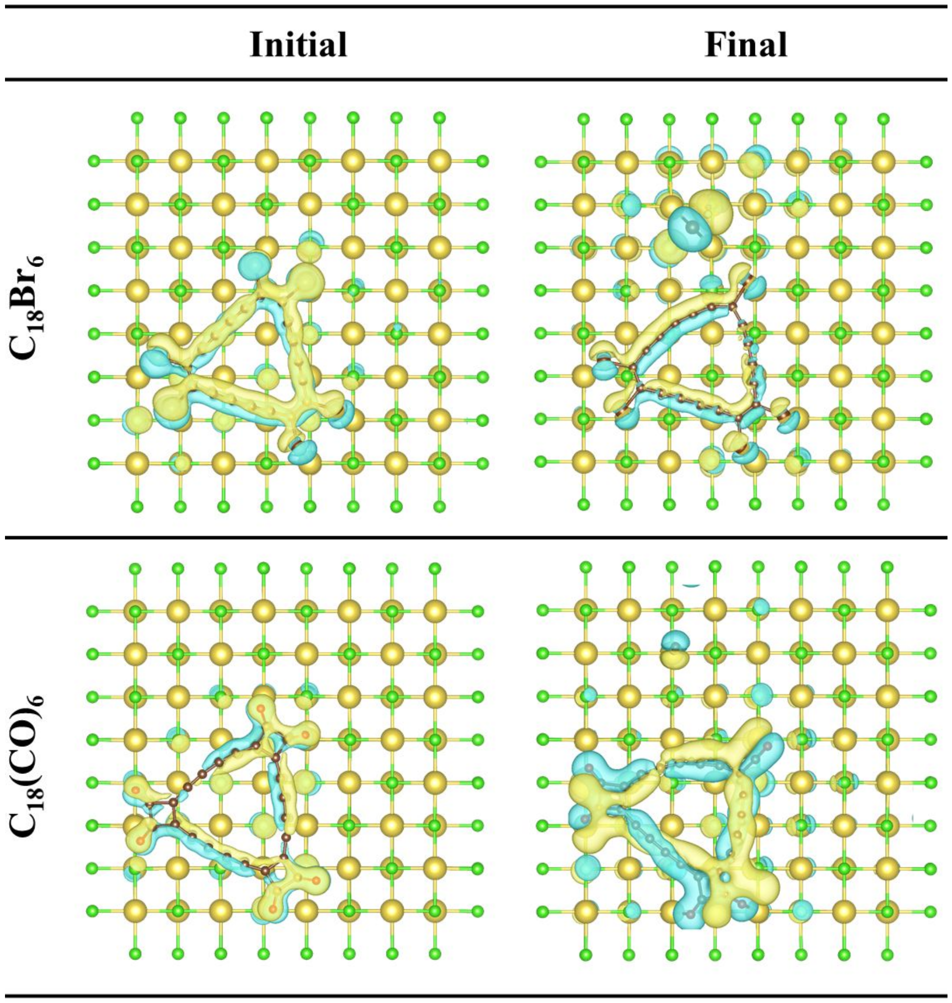

Figure S2: Calculated charge density difference plot of $C_{18}Br_6$ and $C_{18}(CO)_6$ with respect to their anion radicals. The blue distribution corresponds to electrons and the yellow one corresponds to holes. The isosurface level is set to 0.01 e/Å^3. The shift in the spatial density distribution at the molecules can be interpreted by different equilibrium positions of neutral and charged species on the NaCl surface.

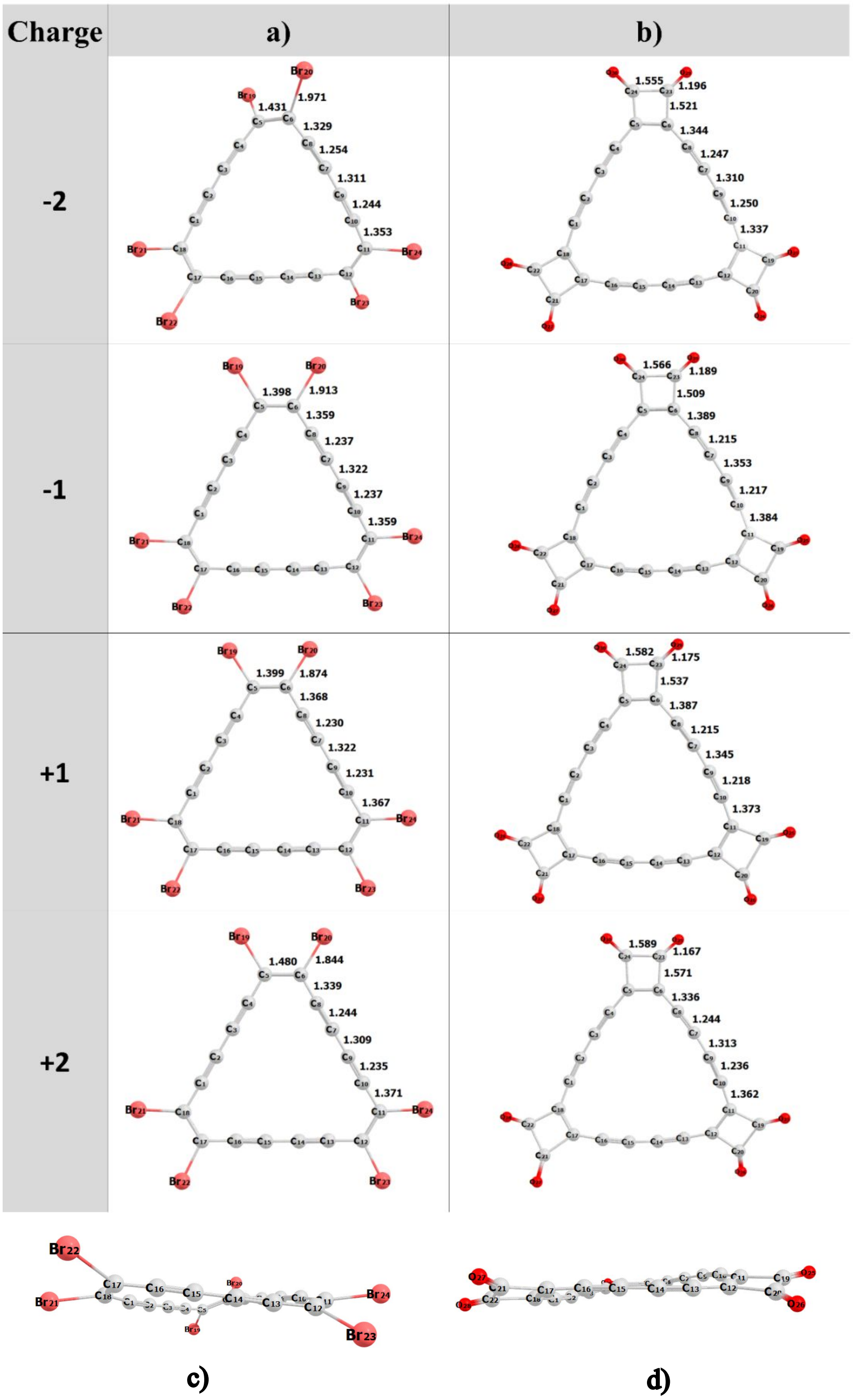

Figure S3. The structures of the a) $C_{18}Br_6$ and b) $C_{18}(CO)_6$ in different charged states. The side view of c) double anion $C_{18}Br_6$ and d) double cation $C_{18}(CO)_6$ optimized at the wB97XD/6-311g(d,p) level of theory.

References

(1) Henkelman, G.; Uberuaga, B. P.; Jónsson, H. A Climbing Image Nudged Elastic Band Method for Finding Saddle Points and Minimum Energy Paths. *J. Chem. Phys*. **2000**, *113*, 9901-9904.

(2) Kresse, G.; Hafner, J. Ab Initio Molecular Dynamics for Liquid Metals. *Phys. Rev. B*. **1993**, *47*, 558–561.

(3) Kresse, G.; Furthmüller, J. Efficiency of Ab-Initio Total Energy Calculations for Metals and Semiconductors Using a Plane-Wave Basis Set. *Comput. Mater. Sci.* **1996,** *6*, 15–50.

(4) Kresse, G.; Hafner, J. Ab Initio Molecular-Dynamics Simulation of the Liquid-Metalamorphous- Semiconductor Transition in Germanium. *Phys. Rev. B.* **1994**, *49*, 14251–14269.

(5) Kresse, G.; Furthmüller, J. Efficient Iterative Schemes for Ab Initio Total-Energy Calculations Using a Plane-Wave Basis Set. *Phys. Rev. B.* **1996**, *54*, 11169–11186.

(6) Perdew, J. P.; Burke, K.; Ernzerhof, M. Generalized Gradient Approximation Made Simple. *Phys. Rev. Lett*. **1996**, *77*, 3865–3868.

(7) Blöchl, P. E. Projector Augmented-Wave Method. *Phys. Rev. B*. **1994**, *50*, 17953–17979.

(8) Grimme, S.; Antony, J.; Ehrlich, S.; Krieg, H. A Consistent and Accurate Ab Initio Parametrization of Density Functional Dispersion Correction (DFT-D) for the 94 Elements H-Pu. *J. Chem. Phys*. **2010**, *132*, 154104.

(9) Momma, K.; Izumi, F. VESTA 3 for Three-Dimensional Visualization of Crystal, Volumetric and Morphology Data. *J. Appl. Crystallogr*. **2011,** *44*, 1272–1276.
