# Supplementary material for: Cyclo[18]carbon Formation from C18Br6 and C18(CO)6 Precursors
Source: J Phys Chem Lett. 2022 Oct 28;13(44):10318–25. doi: 10.1021/acs.jpclett.2c02659 (PMC9661529; doi:10.1021/acs.jpclett.2c02659)
Supplement: Supplementary file 2 — jz2c02659_si_002.pdf [file jz2c02659_si_002.pdf]

jz-2022-02659b.R1

Name: Peer Review Information for "Cyclo[18]carbon Formation from $C_{18}Br_6$ and $C_{18}(CO)_6$ Precursors"

First Round of Reviewer Comments

Reviewer: 1

Comments to the Author

Cyclo[18]carbon has gained considerable attentions in recent years. Although it is already well-known that cyclo[18]carbon can be produced by means of STM tip induced debromination and decarbonylation from its precursors C18Br6 and C18(CO)6, the underlying mechanism has not been explored yet. The current work is interesting, which nicely demystified the voltage induced debromination and decarbonylation mechanisms based on density functional theory calculation. This study was conducted in a reasonable and convincing manner, and the writing is satisfactory. Overall, this is a valuable contribution and I recommend publishing it after a few revisions.

1 PBE was employed for studying adsorption and dissociation of C18Br6 and C18(CO)6 on NaCl surface. However, it is already known that all pure DFT functionals are failed to represent electronic and geometric of cyclo[18]carbon, and at the meantime, these functionals show remarkable delocalization error, which often bring significant error in representing anionic species. How do the authors justify the use of PBE in the current study? The rationale for choosing PBE-D3 should be clarified in the article.

2 It is valuable to study distribution of excessive electron for anionic radical C18Br6 and C18(CO)6 by plotting a density difference map with respect to neutral state, for both initial state and final state geometries. Such maps will provide important complementary information to the ELF map (Fig. 3).

3 The definition of the reaction coordinate exhibited in Fig. 2 should be better clarified, and the choice of the zero point should be mentioned.

4 LUMO is ambiguous for unrestricted DFT calculation, namely it is different for alpha and beta spins. Therefore, the LUMO involved in section "Electronic properties of the C18Br6 and C18(CO)6 molecules and their anion-radical states" should be clearly defined first.

5 V is an unit of voltage rather than that of electric field, therefore some statements such as "...applied electric field of 0.025 V" should be corrected.

6 Direction of external magnetic field should be clearly indicated in Fig. 4 or its figure caption. The AICD isovalue should also be mentioned.

7 Upper and lower limits should be labelled on the color bar in Fig. 3.

8 This statement is somewhat confusing: "The precursor molecules are placed in a way leaving sufficient space on the NaCl surface for the bond dissociation to take place". The meaning of "leaving sufficient space" is quite ambiguous, I suggest the authors use a clearer description instead.

9 Page 13, line 24: cycly[18]carbon should be cyclo[18]carbon

10 "Koopman's theorem" should be "Koopmans' theorem"

Reviewer: 2

Comments to the Author

In the manuscript "Cyclo[18]carbon Formation From C18Br6 and C18(CO)6 Precursors" Suresh and co-workers present an interesting computational study of the formation of cyclo[18]carbon from two precursors with resulting in agreement with experimental observations. I recommend that the paper be accepted for publication after the authors address the minor comments below.

Minor Comments

(1) In the Introduction "The C18Br6 and C18(CO)6 molecules are placed at an optimum distance of ~3.30 Å between the surface and the precursor molecule." How is 3.3 A optimal? Is this from experiment or geometry optimization within DFT?

(2) Choice of DFT Functional: Are the main conclusions invariant to changes in the DFT functional? Do the authors have data with another functional?

(3) Is the use of a NaCl monolayer a computational approximation? Could the NaCl crystal structure (beyond the surface) influence the process?

Author's Response to Peer Review Comments:

Dear Professor Editor,

Thank you very much for your kind consideration of our manuscript. We also would like to thank the referees for their valuable comments and the high rates given to our work. We revised the manuscript following your and the referees' suggestions. The detailed responses can be found in the attached file.

Dear Professor Editor,

Thank you very much for your kind consideration of our manuscript. We also would like to thank the referees for their valuable comments and high rates given to our work. We revised the manuscript following yours and the referees' suggestions. All changes made in the text are highlighted by blue color.

Editor's comment:

1) Supporting Information Statement: A brief, nonsentence description of the actual contents of each supporting information file is required. This description should be labeled Supporting Information and should appear before the Acknowledgement and Reference sections. Examples of sufficient and insufficient descriptions are as follows:

*Examples of sufficient descriptions: "Supporting Information: 1H NMR spectra for all compounds" or "Additional experimental details, materials, and methods, including photographs of experimental setup".

*Examples of insufficient descriptions: "Supporting Information: Figures S1-S3" or "Additional figures as mentioned in the text".

Response: We have included the details about the supporting information in the manuscript as mentioned.

Supporting Information

Computational details, structural parameters of neutral and charged $C_{18}Br_6$ and $C_{18}(CO)_6$ precursor molecules, charge density difference map of both initial and final state of precursor molecules reflecting the difference between neutral and anion-radical states.

2) TOC Graphic: Provide a TOC image per journal guidelines (2 in x 2 in; on the same page as the abstract) with the heading "TOC Graphic" above the graphic. The graphic should be in the form of a structure, graph, drawing, photograph, or scheme—or a combination. Non-scientific cartoon-like images or caricatures are discouraged.

https://pubsapp.acs.org/paragonplus/submission/toc_abstract_graphics_guidelines.pdf

Response to the comment: The TOC is made as per the guidelines and is added to the manuscript alongside the abstract.

3) Title: In both the main manuscript file and the Supporting Information, set the title in title case, with the first letter of each principal word capitalized.

Response: Thank you for the advice. The font of the title in the manuscript and the supplementary information is changed to title case.

4. Title and Author Lists: Title, author names, and affiliations must match in three places: (1) manuscript file, (2) supporting information, and (3) ACS Paragon Plus

Response to the comment: The author names and affiliations are correct and the same in all three places.

5. Abstract: Please label your Abstract and shorten to 150 words or fewer.

Response: The abstract is shortened to 150 words and labeled as suggested.

6. References: In both the main file and the supporting information, fix the style of all references to use JPCL formatting (check all references carefully). ***JPC Letters reference formatting requires that journal references should contain: () around numbers, author names, article title (titles entirely in title case or entirely in lower case), abbreviated journal title (italicized), year (bolded), volume (italicized), and pages (first-last). Book references should contain author names, book title (in the same pattern), publisher, city, and year. Websites must include date of access.

Response: The references have been corrected according to JPCL formatting.

Reviewer(s)' Comments to Author:

Reviewer: 1

Recommendation: This paper is publishable subject to minor revisions noted. Further review is not needed.

Comments:

Cyclo[18]carbon has gained considerable attentions in recent years. Although it is already well-known that cyclo[18]carbon can be produced by means of STM tip induced debromination and decarbonylation from its precursors $C_{18}Br_6$ and $C_{18}(CO)_6$, the underlying mechanism has not been explored yet. The current work is interesting, which nicely demystified the voltage induced debromination and decarbonylation mechanisms based on density functional theory calculation. This study was conducted in a reasonable and convincing manner, and the writing is satisfactory. Overall, this is a valuable contribution and I recommend publishing it after a few revisions.

1) PBE was employed for studying adsorption and dissociation of $C_{18}Br_6$ and $C_{18}(CO)_6$ on NaCl surface. However, it is already known that all pure DFT functionals are failed to represent electronic and geometric of cyclo[18]carbon, and at the meantime, these functionals show remarkable delocalization error, which often bring significant error in representing anionic species. How do the authors justify the use of PBE in the current study? The rationale for choosing PBE-D3 should be clarified in the article.

Response: We thank the referee for this very important note. We agree with the opinion of the reviewer. We should note that in contrast to the cyclo[18]carbon molecule where large HF exchange is typically required to reproduce structural features including bond length alteration, the precursor molecules can be well reproduced within PBE. The studied systems contain more than 200 atoms that also make the application of a hybrid functional computationally inefficient. However, the combination of PBE and D3 correction could give us accurate and relevant results in terms of structural and adsorption features. The use of PBE functional in this study is validated by comparing the bond lengths in the precursor molecules between the present calculation and the hybrid wB97XD/6-311g(d,p) functional which are well in agreement with each other. We have included a figure below comparing the bond lengths between the PBE functional and wB97XD/6-311g(d,p) functional (in parenthesis). One can see that the difference in bond lengths is only ~0.03 Å and that bond length alteration is preserved. The use of Grimme's dispersion D3 correction is described in the computational details section in the ESI. The dispersion interactions between the precursor molecules and the NaCl surface

were accounted for by using Grimme's D3 correction in order to understand if the NaCl surface includes any considerable effect on the adsorption of the precursor molecules.

Though there are no covalent bonds between inert NaCl surface and precursors, it is revealed that the D3 correction can catch the weak interactions affecting the geometries of precursors on the surface which is in line with experimental data.

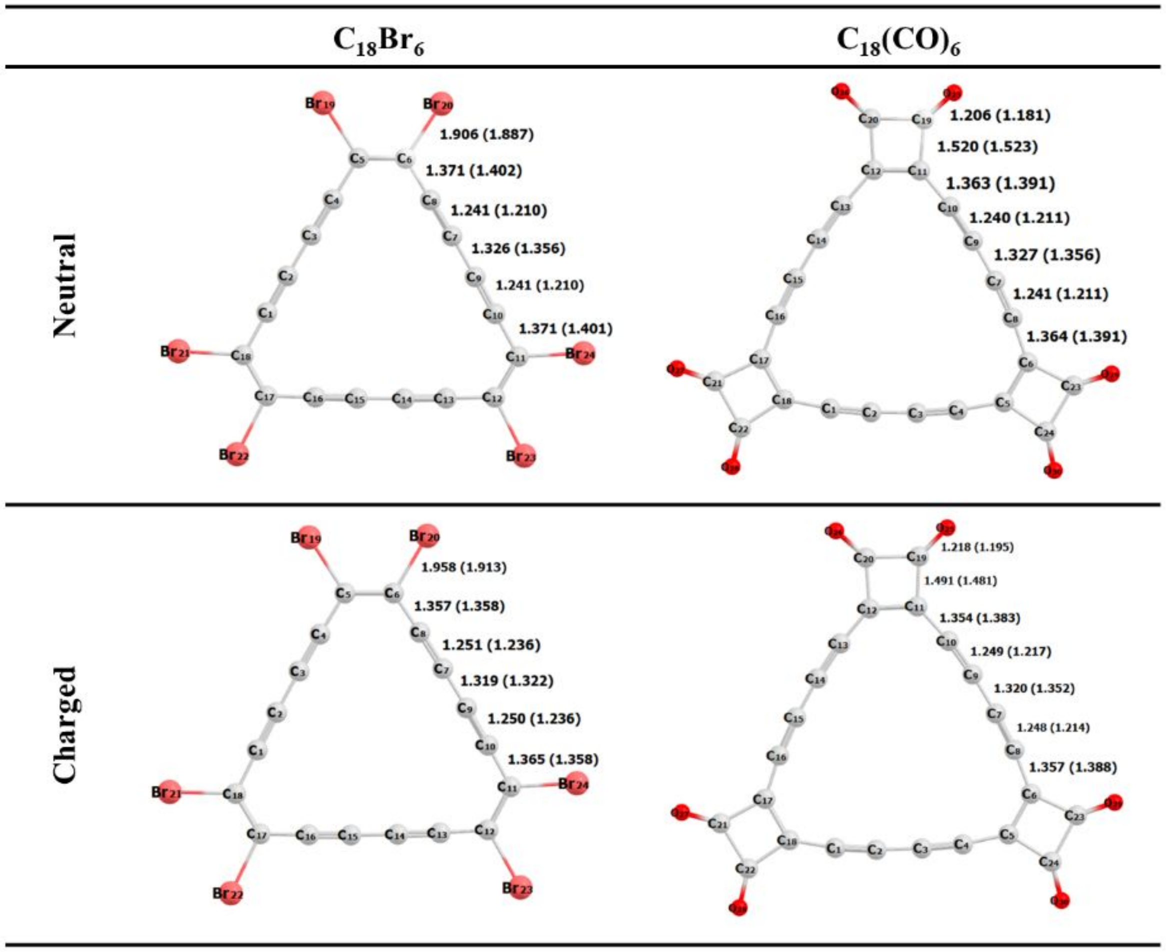

Figure R1: Calculated bond lengths for neutral and charged $C_{18}Br_6$ and $C_{18}(CO)_6$ with PBE-D3 and wB97XD/6-311g(d,p) (in parenthesis) levels of theory.

2) It is valuable to study distribution of excessive electron for anionic radical $C_{18}Br_6$ and $C_{18}(CO)_6$ by plotting a density difference map with respect to neutral state, for both initial state and final state geometries. Such maps will provide important complementary information to the ELF map (Fig. 3).

Response: The calculated density difference map is included in the ESI (Figure S2). And arespecteivediscussion is given.

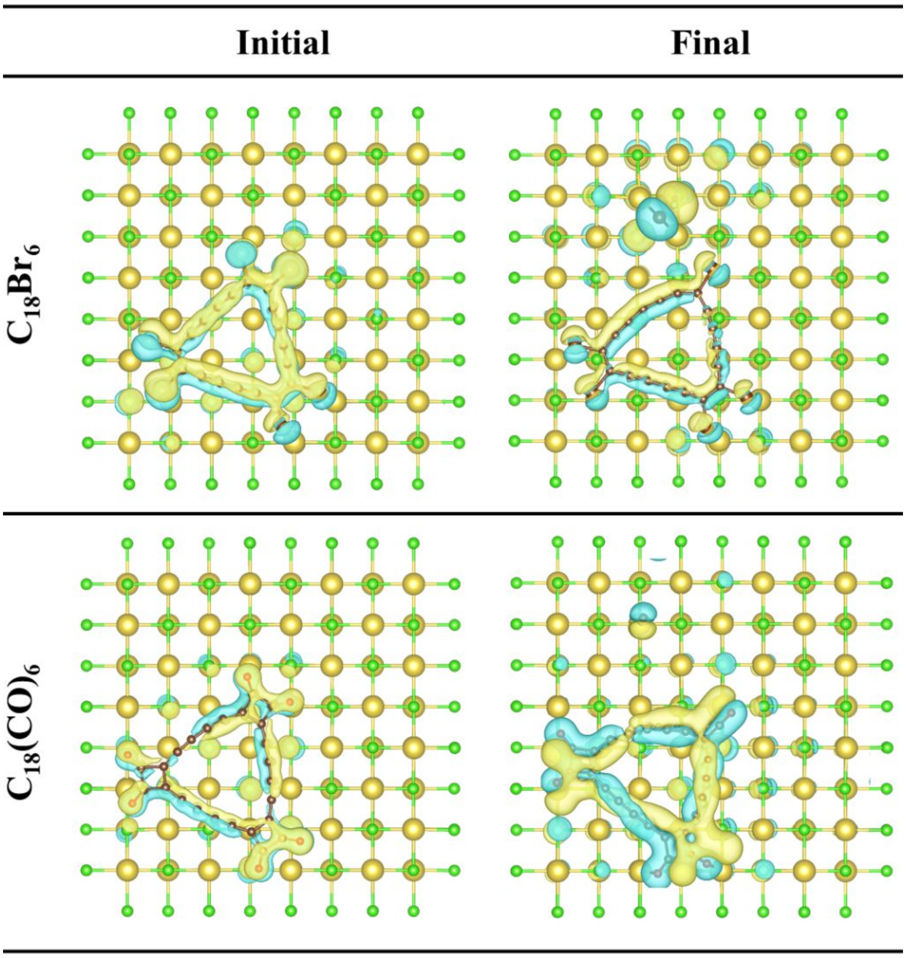

Figure S2: Calculated charge density difference plot of $C_{18}Br_6$ and $C_{18}(CO)_6$ with respect to their anion radicals. The blue distribution corresponds to electrons and the yellow one corresponds to holes. The isosurface level is set to 0.01 e/Å^3. The shift in the spatial density distribution at the molecules can be interpreted by different equilibrium positions of neutral and charged species on the NaCl surface.

In order to visualize the excessive charge effect in the anion state, we calculated the charge density difference plot between the anionic and neutral states of the precursor molecules (Figure S2). Comparing the charge redistribution between the final state of $C_{18}Br_6$ and $C_{18}(CO)_6$, the plot shows higner charge localization on the isolated Br atoms than on the isolated CO species in agreement with the Bader charge analysis.

3) The definition of the reaction coordinate exhibited in Fig. 2 should be better clarified, and the choice of the zero point should be mentioned.

Response: Thank you for the valuable comments. We have included the information in the title description of Figure 2 as follows: The reaction coordinate refers to the images interpolated

between the initial (ground) state "0" and the final state "9". Each intermediate image is minimized in energy along the reaction path while keeping equal spacing with neighboring images.

4) LUMO is ambiguous for unrestricted DFT calculation, namely it is different for alpha and beta spins. Therefore, the LUMO involved in section "Electronic properties of the C18Br6 and C18(CO)6 molecules and their anion-radical states" should be clearly defined first.

Response: We considered the alpha spin state for the calculation and included description to the manuscript as: "The localizations of the highest (or singly) occupied molecular orbitals (HOMO/SOMO) and lowest unoccupied molecular orbitals (LUMO) in spin- α states are shown in Figure 5." and also to the caption of Figure 5 as The α spin state is considered for both SOMO and LUMO levels in case of anion-radical species.

5) V is an unit of voltage rather than that of electric field, therefore some statements such as "...applied electric field of 0.025 V" should be corrected.

Response: The necessary changes have been made and the units of the electric field are denoted as "eV" throughout the manuscript including Figure 2.

6) Direction of external magnetic field should be clearly indicated in Fig. 4 or its figure caption. The AICD isovalue should also be mentioned.

Response: The isosurface value and direction of magnetic field have been added to the caption of Figure 4 as: The direction of the magnetic field is perpendicular to the plane of the molecules. The isosurface value is set to 0.05 a.u.

7) Upper and lower limits should be labelled on the color bar in Fig. 3.

Response: Thank you for the note. We have made the necessary changes in Figure 3.

8) This statement is somewhat confusing: "The precursor molecules are placed in a way leaving sufficient space on the NaCl surface for the bond dissociation to take place". The meaning of "leaving sufficient space" is quite ambiguous, I suggest the authors use a clearer description instead.

Response: As per the reviewer's suggestion, we have made the changes to the manuscript.

In order to simulate dissociated state, the –Br and –CO species were placed at a sufficient distance from the precursor molecule to avoid any spurious interactions and then an energy minimum was found.

Moreover, in the next paragraph, the exact values are mentioned explicitly as: The distance between the dissociated atom/molecule from the initial structure of $C_{18}Br_6$ and $C_{18}(CO)_6$ is 5.55 and 6.01 Å, respectively.

9) Page 13, line 24: cycly[18]carbon should be cyclo[18]carbon

Response: Thank you for pointing out the typo. We have made the respective corrections in the manuscript.

10) "Koopman's theorem" should be "Koopmans' theorem"

Response to the comment: The corrections have been made in the manuscript.

Reviewer: 2

Recommendation: This paper is publishable subject to minor revisions noted. Further review is not needed.

Comments:

In the manuscript "Cyclo[18]carbon Formation From C18Br6 and C18(CO)6 Precursors" Suresh and co-workers present an interesting computational study of the formation of cyclo[18]carbon from two precursors with resulting in agreement with experimental observations. I recommend that the paper be accepted for publication after the authors address the minor comments below.

Minor Comments

1) In the Introduction "The C18Br6 and C18(CO)6 molecules are placed at an optimum distance of ~3.30 Å between the surface and the precursor molecule." How is 3.3 A optimal? Is this from experiment or geometry optimization within DFT?

Response: We thank the referee for the note. The "optimal" distance is just an initial guess which was based on the average atomic radii between the NaCl surface and the precursor molecules. The molecule was placed at different positions on the surface, then it was optimized

without any constraints and the most energetically favorable position was chosen for further calculations.

To clarify this, we, therefore, modified the text as follow: The $C_{18}Br_6$ and $C_{18}(CO)_6$ molecules are initially placed in different positions on the NaCl surface at the distance of ~3.30 Å between the surface and the precursor molecule and then optimized. The most energetically favorable position is adopted for further calculations.

(2) Choice of DFT Functional: Are the main conclusions invariant to changes in the DFT functional? Do the authors have data with another functional?

Response: For PBC calculations of the precursors on the NaCl surface we decided to use the PBE D3 corrected functional by the following reasons: 1) It was important to take into account surface effect because it affects the geometry of precursors which strongly interact with the substrate as compared to C_{18} molecule; 2) the supercell used for calculations contained more than 200 atoms, therefore the application of a hybrid functional becomes inefficient; 3) the results of PBE are validated by the hybrid wB97XD/6-311g(d,p) functional which confirmed its high accuracy in cyclo[18]carbon calculations. The respected data are provided in response 1) to Reviewer 1. This functional is also used in the manuscript to determine the properties of the isolated precursors in both neutral and anion-radical states including calculations of the electronic structures.

(3) Is the use of a NaCl monolayer a computational approximation? Could the NaCl crystal structure (beyond the surface) influence the process?

Response: The choice of NaCl surface is based on the previous experimental data (Reference 1&2). From experimental observations, the dissociated Br atoms and precursor molecules are immobile on the surface indicating a strong interaction between the surface and the precursor molecules which facilitated the high-resolution imaging. From our calculations (in agreement with the experimental data in the mentioned references), we found that the CO groups in the $C_{18}(CO)_6$ molecule interact with the NaCl surface and the precursor loses its planarity. Though NaCl acts as an inert surface for the reaction to occur, the role of the substrate in enhancing the reaction is still in our research interest. However, NaCl does not have any influence on the properties of cyclo[18]carbon as explained in our previous study (reference 3).

1. Kaiser, K.; Scriven, L. M.; Schulz, F.; Gawel, P.; Gross, L.; Anderson, H. L. An Sp-Hybridized Molecular Carbon Allotrope, Cyclo[18]Carbon. *Science.* **2019**, *365* (6459), 1299–1301. https://doi.org/10.1126/science.aay1914

2. Scriven, L. M.; Kaiser, K.; Schulz, F.; Sterling, A. J.; Woltering, S. L.; Gawel, P.; Christensen, K. E.; Anderson, H. L.; Gross, L. Synthesis of Cyclo[18]Carbon via Debromination of C18Br6. J. Am. Chem. Soc. 2020, 142 (30), 12921–12924. https://doi.org/10.1021/jacs.0c05033.

3. Baryshnikov, G. V.; Valiev, R. R.; Kuklin, A. V.; Sundholm, D.; Ågren, H. Cyclo[18]Carbon: Insight into Electronic Structure, Aromaticity, and Surface Coupling. J. Phys. Chem. Lett. 2019, 10 (21), 6701–6705. https://doi.org/10.1021/acs.jpclett.9b02815.
